# Spectrophotometric, Spectrofluorimetric and Voltammetric Analyses of Naftidrofuryl Oxalate in its Tablets

**Published:** 2009-09

**Authors:** Suzy M. Sabry, Tarek S. Belal, Magda H. Barary, Mohammed E. A. Ibrahim

**Affiliations:** *Department of Pharmaceutical Analytical Chemistry, Faculty of Pharmacy, University of Alexandria, Elmessalah 21521, Alexandria, Egypt*

**Keywords:** naftidrofuryl oxalate, derivative spectrophotometry, spectrofluorimetry, differential-pulse voltammetry, pharmaceutical tablets

## Abstract

This work deals with several direct and indirect spectrophotometric, spectrofluorimetric and voltammetric analyses of the vasodilator drug: naftidrofuryl oxalate (NF). For the derivative spectrophotometric measurement, NF was determined by measuring the peak to peak amplitude of ^1^D_263–299_ and ^2^D_282–311_ or the absolute peak height of ^1^D_237_ and ^2^D_241_, while its reaction product with concentrated sulfuric acid was determined by measuring the peak to peak amplitude of ^2^D_248–263_ or the absolute peak height of ^1^D_275_. For the spectrofluorimetric measurement, native NF fluorescence was measured in Britton-Robinson buffer (pH 5) at λ_em_ = 331 nm (λ_ex_ = 277 nm), while the reaction product was measured in aqueous solution at λ_em_ = 385 nm (λ_ex_ = 258 nm). All factors affecting these analyses were studied and optimized. This work also describes a differential pulse cathodic voltammetric determination of the NF reaction product with concentrated sulfuric acid at the hanging mercury drop electrode (HMDE) where the experimental conditions affecting analysis including buffer pH, pulse amplitude and scan rate were examined and optimized. The chemical structure of the reaction product with concentrated sulfuric acid was investigated using several spectroscopic methods. All the developed procedures were validated and satisfactorily applied for the determination of NF in its pharmaceutical tablets.

## INTRODUCTION

Naftidrofuryl oxalate (NF) is also known as nafronyl oxalate, (2-(diethylaminoethyl)-2-[(naphthalene-1-yl)methyl]-3-(tetrahydrofuran-2-yl)propanoate hydrogen oxalate) (1). It is used as a vasodilator in the treatment of peripheral and cerebral vascular disorders. It is claimed to enhance cellular oxidative capacity thereby protecting cells against the results of ischaemia (2). The BP 2008 (1) specifies a potentiometric non aqueous titration and HPLC methods for the assay of NF in bulk form and capsules, respectively.

Few analytical methods have been reported for the determination of NF in biological fluids and/or pharmaceutical preparations. Most of these studies focused on HPLC-fluorimetric (3–6) and HPLC-UV detection methods (7, 8) and phosphorimetric analysis (9–12). Others include a potentiometric method with nafronyl ion-selective electrodes (13), flow injection analysis with fluorescence optosensor (14) and a spectrophotometric method (15).

**Figure 1:**
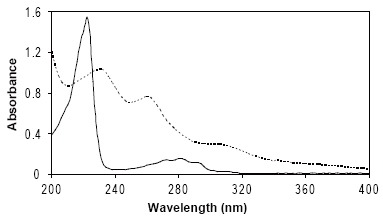
Absorption spectra of 10 μg/mL NF (——) and PHN (≡10 μg/mL NF) (---), both in sulfuric acid.

NF is a naphthalene derivative, thus it has its own UV spectral features including a sharp absorption peak at 222 nm which is strongly absorbing (ε=73171.2) and a broad absorption peak at 282 nm which is weakly absorbing (ε=7577.6) (Figure 1). Additionally, such a rigid hydrocarbon skeleton (naphthalene) shows native luminescence characters. Previous reports have been focused on establishment of different phosphorimetric analytical procedures including; heavy-atom induced room-temperature phosphorimetry (9, 10) and micellar-stabilized heavy-atom induced room-temperature phosphorimetry (11, 12). Only single spectrophotometric method (15) has been reported which was based on absorbance measurement of NF at 283 nm in saline solution. To the best of our knowledge, no study handles simple and direct derivative spectrophotometry or even direct spectrofluorimetric assay of NF, has been reported. Also no polarographic or voltammetric method has been described as NF exhibits no electro-activity at mercury electrode surface.

Two main goals were considered in the present work. First, to develop simple derivative-spectrophotometric and spectrofluorimetric methods of analysis based on direct measurement of ^1^D_237_, ^1^D_263–299_, ^2^D_241_ and ^2^D_282–311_ of NF in 0.05 M sulfuric acid and measurement of native fluorescence emission intensity at λ_em_ = 331 nm (λ_ex_ = 277 nm) in Britton-Robinson (B-R) buffer of pH 5. Second, is to establish sensitive voltammetric method of analysis of NF. Consequently, the structural features of NF molecule were thought to be exploited to design an electro-active site via its reaction with concentrated sulfuric acid. The reaction offers an electro-active site (C=O, Ketonic group) with cathodic activity at mercury electrode. Moreover, dramatic changes in UV spectral- and fluorescence emission-characters were observed as a consequence of chromophoric structural expansion (tri-cyclic skeleton with carbonyl group instead of bi-cyclic naphthalene ring). Accordingly, the study dealing with NF-reaction was channeled through four approaches; (a) isolation and purification of the reaction product and elucidation of the structure by mass spectrometry (MS), ^1^H-NMR and IR spectrometry, (b) optimization of the reaction conditions and other experimental factors to allow maximum analytical sensitivity, (c) study of the voltammetric behavior of the reaction product at HMDE to establish sensitive DP voltammetric method of analysis and (d) development of sensitive derivative-spectrophotometric and spectrofluorimetric methods, as alternative comparative methods, based on measurement of ^1^D_275_ and ^2^D_248–263_ and fluorescence emission intensity at λ_em_ = 385 nm (λ_ex_ = 258 nm) of the reaction product in aqueous acidic solution. The applicability of the proposed methods was evaluated through the determination of NF in pure form and tablets formulation.

## EXPERIMENTAL

### Instrumentation

Spectrophotometric measurements were performed using a Perkin-Elmer, Lambda EZ201 UV-VIS spectrophotometer (Waltham, MA, USA) with matched 1-cm quartz cells. Fluorescence measurements were carried out using a Shimadzu (Kyoto, Japan) RF-1501 version 3.0 spectrofluorophotometer equipped with a 150 W xenon lamp and 1-cm quartz cell. The voltammograms were obtained with a Metrohm 693 VA Processor and a Metrohm 694 VA Stand (Metrohm Ltd, Herisau, Switzerland) was used in the hanging mercury drop electrode (HMDE) mode. The three electrode system was completed by means of a Ag/AgCl (3 M KCl) reference electrode and a Pt auxiliary electrode.

### Materials

Naftidrofuryl Oxalate (98%) was kindly provided by Minapharm Ind., Cairo, Egypt. Analytical grade of sulfuric acid, orthophosphoric acid, boric acid, acetic acid, sodium hydroxide, ethanol, isobutanol and high purity distilled water were used in the study. Pharmaceutical preparation examined in this study is Praxilene^®^ tablets (Minapharm Ind., Cairo, Egypt under license of Merck Santé France, BN. 5AE0028) labeled to contain 200 mg naftidrofuryl oxalate per tablet.

## METHODS

### Preparation of reference standard solutions of NF

Standard solutions of NF (100 μg/mL) were prepared, in 0.05 M sulfuric acid (for derivative spectrophotometric determination), or in distilled water (for spectrofluorimetric determination) or in ethanol (for the reaction with concentrated sulfuric acid), and kept refrigerated at 4°C. Working standard solutions were prepared by suitable dilution steps with the appropriate solvent in accordance with the concentration range used in the analytical technique followed.

### Preparation of tablets assay solutions

A total of 20 tablets (Praxilene^®^ tablets) were massed and finely powdered. To an accurately weighed quantity of the powder equivalent to 10 mg NF, 60 mL of the corresponding solvent (0.05 M sulfuric acid for direct derivative-spectrophotometry or distilled water for direct spectrofluorimetry or ethanol for sulfuric acid reaction) were added, stirred for 10 minutes then filtered into a 100-mL volumetric flask. The residue was washed with two 10 mL portions of the same solvent and the washing solutions were added to the filtrate and diluted to volume with the corresponding solvent. This solution, claimed to contain 100 μg/mL of NF, was used for the assay procedure after suitable dilution steps with the appropriate solvent in accordance with the concentration range used in the analytical technique followed.

### Procedure for direct derivative spectrophotometric and spectrofluorimetric analyses of NF

The first and second UV derivative spectra were recorded for standard/assay NF solutions within the concentration ranges listed in Table 1 in 0.05 M sulfuric acid. The ^1^D_237_, ^1^D_263–299_ (absolute value for the peak to trough amplitude of the ^1^D spectrum between 263 and 299 nm), ^2^D_241_ and ^2^D_282–311_ (absolute value for the peak to trough amplitude of the ^2^D spectrum between 282 and 311 nm) amplitudes were measured. The fluorescence emission intensity was measured for standard/assay NF solutions within the concentration range of 0.01–0.3 µg/mL (Table 1) in B-R buffer (Composed of a mixture of phosphoric, boric and acetic acids) of pH 5, at λ_em_ = 331 nm (λ_ex_ = 277 nm).

### Preparation of concentrated sulfuric acid reaction solutions

Suitable separate aliquots of ethanolic NF standard/assay solution were evaporated to dryness in a boiling water bath. Separate volumes (0.5 mL each) of concentrated sulfuric acid were added to the residues. The resultant reaction solutions were heated in a boiling water bath for 50 minutes.

### Procedure for voltammetric analysis of NF-concentrated sulfuric acid reaction product [2-(tetrahydrofuran-2-yl-methyl)-2,3-dihydro-1*H*-phenalen-1-one], PHN

Aliquots of the reaction solution (standard/assay) giving final concentration range of 1–20 μg/mL, were neutralized with sodium hydroxide (10 M) and made to volume with B-R buffer to give a final pH, 11. Then the solution was transferred into the voltammetric cell and purged with nitrogen gas for 5 min. The voltammograms were recorded between −500 to −1200 mV using the HMDE. Unless otherwise stated, the following parameters were used; −100 mV pulse amplitude, 10 mV s^−1^ scan rate, 4 mV potential interval, a drop size of ca. 0.6 mm^2^ drop area, constant stirrer speed of 2000 rpm.

### Procedure for derivative spectrophotometric and spectrofluorimetric analyses of PHN

Aliquots of the reaction product (standard/assay) were completed to the volume with distilled water to obtain final concentration ranges of 0.0025–0.06 µg/mL (spectrofluorimetry) and 0.5–20.0 µg/mL (derivative-spectrophotometry). The fluorescence intensities were measured at λ_em_ = 385 nm (λ_ex_ = 258 nm). The first and second UV derivative spectra were recorded and the ^1^D_275_ and ^2^D_248–263_ amplitudes were measured. It should be noted that in all the techniques, the diluting solvent was added to the reaction solution gradually whilst keeping the reaction mixture in an ice-water bath.

### Preparation and isolation of PHN for chemical structure confirmation

A quantity of NF (500 mg) was mixed with 2 mL of concentrated sulfuric acid. The reaction mixture was then heated in a boiling water bath for 50 minutes, then cooled and diluted with ∼5 mL water. The reaction solution was twice extracted with 15 mL portion of isobutanol. The combined isobutanol extract was dried over anhydrous sodium sulphate and then evaporated to dryness under vacuum leaving a reddish brown solid residue.

## RESULTS AND DISCUSSION

### Derivative-spectrophotometric and fluorimetric analyses of NF

**Figure 2:**
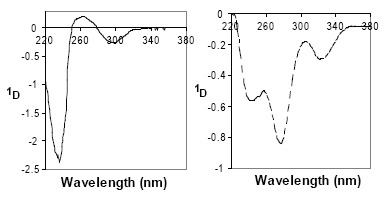
First derivative spectra (Δλ = 6 nm) of 10 μg/mL NF (—) and PHN (≡ 10 μg/mL NF) (----), both in sulfuric acid.

**Figure 3:**
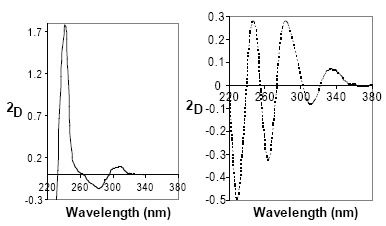
Second derivative spectra (Δλ = 6 nm) of 10 μg/mL NF (——) and PHN (≡ 10 μg/mL NF) (----), both in sulfuric acid.

The naphthalene moiety of NF molecule offers a characteristic UV absorption spectrum as well as a luminescence property. Figure 1 shows the zero-order UV spectrum of NF in 0.05M sulfuric acid. It has absorption maxima at 222 nm with molar absorptivity, ε, equals 73171.2 (log ε=4.86) and at 282 nm with molar absorptivity, ε, equals 7577.6 (log ε=3.88). The first- and second-derivative UV spectra of NF exhibit several maxima and minima at different wavelengths (Figures 2 and 3). The first- and second-derivative spectrophotometric techniques (^1^D_237_, ^1^D_263–299_, ^2^D_241_ and ^2^D_282–311_ measurements) were suggested for the assay of NF.

**Figure 4:**
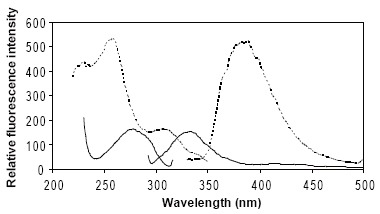
Excitation and emission spectra of 0.05 μg/mL NF in B-R buffer pH 5 (—) and PHN (≡ 0.05 μg/mL NF) in aqueous acidic solution (----).

NF showed characteristic excitation and emission spectra presented in Figure 4. It exhibits native fluorescence emission at 331 nm, in acidic buffer solution of pH 5 (λ_ex_ = 277 nm). To achieve better sensitivity, the fluorescence intensity of NF in different diluting solvents, including B-R buffer of different pH values, distilled water, 0.1M NaOH, acetonitrile, dimethylformamide, ethanol, methanol and dioxan was studied. Acidic buffer solution was found to be superior. Further study was done to check for the influence of pH of buffer solution on fluorescence intensity. The study indicated best sensitivity at pH 5. The spectrofluorimetric method was based on measurement of native fluorescence emission of NF, in acidic buffer solution, at λ_em_ = 331 nm (λ_ex_ = 277 nm).

### Aspects of NF-sulfuric acid reaction and different methods of analysis

**Figure 5:**
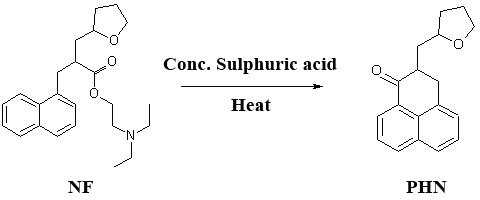
NF-concentrated sulfuric acid reaction.

**NF-sulfuric acid reaction pathway.** Referring to NF molecule, the ester link associates naphthalene propanoic acid and diethylamino ethyl moieties. Stress concentrated sulfuric acid/heat affects the cleavage of ester link simultaneously with cyclization to yield tricyclic keto species, 2-(tetrahydrofuran-2-yl-methyl)-2,3-dihydro-1*H*-phenalen-1-one (PHN). Such a dramatic change in the chromophore structure significantly affects the UV and fluorescence spectral characteristics. More worthy, is the entrance of an electro-active site, the carbonyl moiety (C=O). The following scheme represents the proposed pathway for NF-concentrated sulfuric acid reaction (Figure 5).

**Evidence of NF-sulfuric acid reaction pathway.** The structure of the isolated product was confirmed by IR, ^1^H-NMR and MS spectroscopic methods. All criteria have assigned the structure to be 2-(tetrahydrofuran-2-yl-methyl)-2,3-dihydro-1H-phenalen-1-one, PHN. The IR spectrum (KBr) of PHN refers to C=O stretching peak at 1638 cm^−1^ (that of NF is at 1730 cm^−1^).

The isolated species has the formula, C_18_H_18_O_2_. The EI mass spectrum shows diagnostic fragment ions *m/z* 208 (M^+^ - C_3_H_6_O, 15.4%), *m/z* 163, (C_11_H_15_O, 13.5%), *m/z* 129 (C_9_H_5_O, 46.1%), *m/z* 127 (C_10_H_7_, 26.9%), *m/z* 108 (C_6_H_4_O_2_, 28.8%) and *m/z* 92 (C_6_H_4_O, 76.9%).

The ^1^H-NMR spectrum of PHN (methanol, δ ppm, 500 MHz): 1.27 (d, 2H, CH_2_-methyl bridge); 1.69 (m, 2H, C_4_-H furane); 1.90 (m, 2H, C_3_-H furane); 3.22 (m, H, C_2_-H phenalenone); 3.53 (m, H, C_2_-H furane); 3.73 (d, 2H, C_3_-H phenalenone); 3.81 (t, 2H, C_5_-H furane); 7.5–9.3 (m, Ar-H).

**Figure 6:**
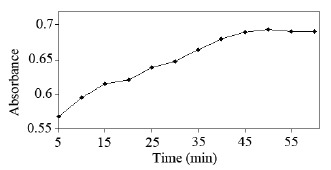
Effect of time course on NF-sulfuric acid reaction (≡ 10 μg/mL NF) at 100°C, at λ = 260 nm.

**Heat-time course optimization of NF-sulfuric acid reaction.** The influence of heat-time course on the reaction sequence was studied, to achieve maximum analytical sensitivity towards voltammetric, derivative-spectrophotometric and spectrofluorimetric analyses. The first preliminary experiments indicated that 100°C is just adequate to activate the reaction. Lower temperature does not meet the rapidity requirement. So, all the studies were carried out at 100°C. The time course of sulfuric acid-NF reaction was followed through spectrophotometric measurement of the reaction product at 260 nm. Figure 6 displays the data in term of A_260_–time profile. The absorbance values reached a plateau close to 50 min.

### Different analytical methods for determination of NF in term of PHN

#### Differential pulse voltammetric determination of PHN

Based on the electro-activity of PHN, a DP voltammetric assay was developed for determination of NF. It is based on measurement of peak current of the voltammetric cathodic peak of the reaction solutions at −1000 mV using the HMDE versus Ag/AgCl electrode in B-R buffer of pH 11. The relevant analytical parameters are listed in Table 2.

#### The electro-active behavior of PHN at HMDE and pH study

**Figure 7:**
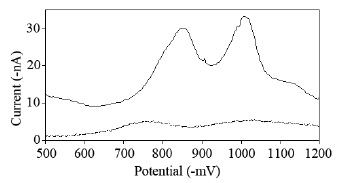
Differential-pulse voltammograms of PHN (≡ 10 μg/mL NF) (—) and B-R buffer pH 11 (----), versus Ag/AgCl reference electrode.

PHN is an electro-active species. The DP voltammogram in B-R buffer of pH 11 is given in Figure 7. It displays two well defined cathodic peaks at −840 mV and at −1000 mV. The latter signal showed higher sensitivity and better linear response with concentration and was used for quantitative work.

Britton–Robinson buffer was chosen as a supporting electrolyte for the DP voltammetric determination of PHN. The effect of pH on the peak current was studied over the pH range 6–12. The cathodic peaks show a noticeable increase in peak current and the *E*p values were shifted to more negative potential upon increasing the pH. At pH<9, a poor voltametric peak was observed at −740 mV for pH 6 and at −770 mV for pH 7. Increasing the pH between 9 to 11 resulted in a remarkable enhancement in peak current then a decline happened at pH12. The optimum pH=11 where a pre-peak was observed at lower potential (−840 mV) and close near to working peak, it showed no linear response. A shift of the peak potential towards more negative value as the pH increases, indicates the involvement of protons in the electrode reaction. According to this investigation, the supporting electrolyte, B-R buffer of pH 11 and voltammetric measurement at −1000 mV were chosen as the most appropriate for the current on-going study.

#### Electrode reaction and reversibility

**Figure 8:**
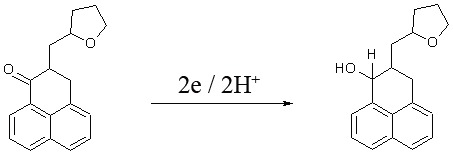
Electrode reaction of PHN

The following scheme shows the electrode reaction of PHN. The electro-active center, C=O, is reduced in a one step of 2e/2H^+^ reaction to hydroxyl group. This is similar to electrochemical reactions of other ketones (16) (Figure 8).

To study the reversibility of the reduction process, the technique reported by Brike *et al* (17) was used. The differential pulse voltammogram was recorded with a positive-going potential pulse (+50 mV) then with a negative-going potential pulse (−50 mV). It was found that the reaction product reduction process corresponds to the quasi-reversible criteria:

$$1)\;{\text{Ep}}^{\text{c}}-{\text{Ep}}^{\text{a}}<|\Delta \text{E}|$$

where Ep^c^ is cathodically scanned potential, Ep^a^ is anodically scanned potential and ΔE is the pulse amplitude.

$$2)\;{\text{Ip}}^{\text{a}}/{\text{Ip}}^{\text{c}}<1$$

where Ip^a^ is current measured at anodically scanned potential and Ip^c^ is current measured at cathodically scanned potential.

These values were Ep^c^ − Ep^a^ = 29 mV (ΔE = 50 mV) and Ip^a^/Ip^c^ = 0.485 (<1).

#### Instrumental parameters

Instrumental conditions affecting the peak current were optimized. Maximum response was obtained at scan rate of 10 mV/s. Also, the effect of the pulse amplitude on the peak current was studied. The plot is linear up to pulse amplitude of −100 mV which is the instrument maximum, therefore it was chosen for analytical measurement.

#### Investigation of the stripping voltammetry performance

In order to enhance the sensitivity of the voltammetric analysis, the stripping voltammetric performance was investigated. Accordingly, the adsorption characteristics of PHN at the mercury electrode was examined over the pH range from 7 to 11, using concentration level 20 µg/mL, the accumulation potential studied ranged from 0 to −1200 mV for a pre-adsorption time of 20 seconds. All trials point out one fact, PHN shows no adsorption phenomenon at the mercury electrode.

#### Derivative-spectrophotometric and fluorimetric determination of PHN

The remarkable change in the chromophore structure of NF significantly affects the UV and fluorescence spectral characteristics. Figure 1 shows the zero-order UV spectrum of PHN in aqueous acidic solution. It has absorption maxima at 232 nm with molar absorptivity, ε equals 26734.5 (log ε=4.43) and at 260 nm with molar absorptivity, ε equals 20398.8 (log ε=4.31). The first- and second-derivative UV spectra of PHN exhibit several maxima and minima at different wavelengths (Figures 2 and 3). The first- and second-derivative spectrophotometric techniques (^1^D_275_ and ^2^D_248–263_ measurements) were suggested for the assay of NF in term of PHN.

Figure 4 displays the excitation and emission spectra of PHN in aqueous solution. A red-shift was observed in the emission peak. To attain best analytical sensitivity, the fluorescence intensity of PHN, in different working solvents, was examined. Solvents tried were distilled water, B-R buffer, 0.1 M NaOH and several organic solvents such as ethanol, methanol, acetonitrile and DMF. Water gave the highest fluorescence intensity and the least background fluorescence. The proposed spectrofluorimetric method was based on measurement of fluorescence emission of PHN, in aqueous acidic solution, at λ_em_ = 385 nm (λ_ex_ = 258 nm). Table 1 represents the analytical parameters for the proposed derivative-spectrophotometric and spectrofluorimetric methods.

**Stability.** The stability of the reaction solutions of NF-sulfuric acid was checked in the working solvents, distilled water (relative fluorescence intensity measurement at λ_em_ = 385 nm (λ_ex_ = 258 nm) and B-R buffer of pH 11 (DP voltammetric measurement at −1000 mV). Up to 3h, no variation was observed.

### Points of consideration in view of comparative analytical methods

Direct spectrophotometry of NF (A_220 nm_ measurement, short wavelength region) may be adversely affected by spectral interference (non-specific irrelevant absorbance, formulation matrix) during analysis of pharmaceutical preparations, while derivative spectrophotometry (^1^D_237_, ^1^D_263–299_, ^2^D_241_ and ^2^D_282–311_ measurements) can overcome background interference.

Indeed, the spectral features of PHN show a noticeable shift in absorption maximum to longer wavelength, in addition to enhancement in absorptivity. This matter seems to be important towards enhanced sensitivity of direct spectrophotometry, A_260_, with minimal formulation matrix interference. Additionally, the derivative technique (^1^D_275_ and ^2^D_248–263_) increases the method selectivity and improves spectral features. The indirect derivative spectrophotometric (^1^D_275_ and ^2^D_248–263_) method achieves better sensitivity (∼6 folds higher) than the direct method (^1^D_263–299_ and ^2^D_282–311_). Even though, the latter, at short wavelength region (^1^D_237_, and ^2^D_241_), records amazing sensitivity.

Owing to structural differences of NF and PHN, the latter shows stronger fluorescence emission at longer wavelength. Accordingly, spectrofluorimetric analysis through NF-sulfuric acid reaction indicates analytical sensitivity ∼3–4 folds higher than direct method (native fluorescence measurement of NF). Finally, the developed direct- and indirect-analytical methods (derivative spectrophotometry and spectrofluorimetry) assure the validity items (specificity, accuracy, precision, linearity, ranges, LOD and LOQ values).

### Validation of the proposed methods

#### Linearity and concentration ranges

Tables 1 and 2 assemble the analytical performance data for the proposed methods. These include regression equations computed from calibration graphs, concentration ranges, correlation coefficients along with the standard deviations of the intercept (S_a_), slope (S_b_) and residuals (S_y/x_). The linearity was also evaluated by calculation of percentage relative standard deviation of the slope (S_b_ %). It was found to be around 2% for all the developed analytical methods. An important statistic for indicating the random error in the estimated values of y is the standard error of the estimate, or standard deviation about regression, or standard deviation of residuals, S_y/x_. The smaller the standard error of the estimate, the closer the points are to the straight line.

**Table 1:** Analytical parameters for the derivative-spectrophotometric and spectrofluorimetric determinations of NF and its reaction product with concentrated sulfuric acid: PHN

Method	Wavelength λ (nm)	Linearity Range (µg/mL)	Regression Equation			S_a_ a	S_b_ b (%S_b_)^c^	S_y/x_ d	LOD^e^ (µg/mL)	LOQ^f^(µg/mL)
			Intercept (a)	Slope (b)	Corr. Coeff. (r)					

Derivative Spectrophotometry (NF)	^1^D_263–299_	3–60	−0.0011	0.0459	0.9999	0.003	0.0001 (0.26)	0.006	0.006	0.018
	^2^D_282–311_	3–60	−0.0002	0.0262	0.9998	0.002	0.0003 (1.03)	0.002	0.002	0.008
	^1^D_237_	0.2–6	−0.0287	0.2393	0.9999	0.003	0.001 (0.48)	0.006	0.001	0.004
	^2^D_241_	0.2–6	−0.0132	0.1818	0.9999	0.002	0.0007 (0.39)	0.003	0.0007	0.003
Spectrofluorimetry (NF)	λ_ex_/λ_em_ (277/331)	0.01–0.3	13.2389	2795.60	0.9994	5.770	40.11 (1.44)	10.53	0.0009	0.003
Derivative spectrophotometry (PHN)	^1^D_275_	0.5–20	−0.0157	0.0834	0.9996	0.016	0.002 (2.0)	0.018	0.085	0.28
	^2^D_248–263_	0.5–20	0.0009	0.0751	0.9999	0.002	0.0004 (0.52)	0.003	0.032	0.11
Spectrofluorimetry (PHN)	λ_ex_/λ_em_ (258/385)	0.0025–0.06	14.7766	11052.56	0.9998	4.620	168.18 (1.52)	5.87	0.00007	0.0002

a Standard deviation of the intercept;

b Standard deviation of the slope;

c Percentage relative standard deviation of the slope;

d Standard deviation of residuals;

e Limit of detection;

f Limit of quantitation.

**Table 2:** Experimental and analytical parameters for the voltammetric determination of PHN

Parameter	Value

Pulse amplitude (mV)	−100
Scan rate (mV sec^−1^)	10
Peak potential (mV)	−1000
Concentration range (μg/mL)	1–20
Regression equation	
Intercept (a)	1.4265
Slope (b)	3.8813
Correlation coefficient (r)	0.9998
S_a_ a	0.35
S_b_ b (% S_b_)^c^	0.062 (1.60)
S_y/x_ d	0.43
LOD^e^ (μg/mL)	0.07
LOQ^f^ (μg/mL)	0.02

a Standard deviation of the intercept;

b Standard deviation of the slope;

c Relative percentage standard deviation of the slope;

d Standard deviation of residuals;

e Limit of detection;

f Limit of quantitation.

#### Specificity

The measured peaks (derivative-spectrophotometry, the spectrofluorimetry and DP voltammetry) of test solutions “Praxilene^®^ tablets” were found to coincide exactly with regard to their position, with the corresponding peaks of the standard solutions.

#### Accuracy

The accuracy of the proposed methods has been tested by preparing mixtures of commonly used tablet adjuvants (glucose, starch, magnesium stearate and cellulose) with different concentrations of analyte (three levels). Mean % recoveries of the added drug using the different analytical methods are presented in Table 3. The results obtained indicate the high accuracy of the proposed methods and no effect of commonly used adjuvants on the determination of the investigated drug.

**Table 3:** Accuracy and precision for the different analytical methods.

Measurement	Nominal Value (mg/mL)	Mean % Recoveries a	RSD (%)

Derivative-spectrophotometry			
^1^D_263–299_ (NF)	5	100.45	2.04
	10	99.60	1.10
	20	97.50	1.65
^2^D_282–311_ (NF)	5	100.95	1.85
	10	100.10	1.40
	20	101.23	0.87
^1^D_237_ (NF)	0.5	98.00	1.10
	1	99.98	1.78
	5	101.40	0.59
^2^D_241_ (NF)	0.5	98.50	1.30
	1	99.90	1.50
	5	100.20	0.80
^1^D_275_ (PHN)	0.5	102.00	1.55
	1	101.00	1.98
	10	98.90	1.01
^2^D_248–263_ (PHN)	0.5	100.50	1.12
	1	101.00	0.99
	10	99.50	1.33
Spectrofluorimetry			
(NF) λ_ex/em_ (277/331 nm)	0.05	100.66	0.56
	0.1	98.42	1.19
	0.2	100.34	0.13
(PHN) λ_ex/em_ (258/385 nm)	0.005	99.40	1.25
	0.01	100.50	0.81
	0.05	102.12	0.76
DP Voltammetry			
(PHN) at −1000 mV	5	99.40	1.61
	10	100.70	0.59
	20	98.45	1.12

a Mean % recovery of five determinations in presence of tablet adjuvants.

#### Precision

The precision, assessed by five replicate determinations at three different concentration levels, has been carried out (Table 3). The percentage relative standard deviation (RSD %) values were found to be within the acceptable limit, 2%.

#### Detection and quantitation limits

The limits of detection, LOD, and of quantitation, LOQ, were calculated in accordance to the formulae given by the official compendia methods [USP method (18)], where LOD and LOQ are defined as 3s.b^−1^ and 10s.b^−1^, respectively (s is the standard deviation of replicate blank responses and b is the slope of the regression equation). Data are given in Tables 1 and 2.

### Analysis of Pharmaceutical Preparation

The proposed methods were applied to the determination of NF in its dosage form. As can be seen from the results shown in Tables 4 and 5, the methods gave satisfactory recovery data for NF. The RSD (%) and mean % found for the assay results showed high reproducibility and accuracy of the proposed methods. Tablet adjuvants and inactive ingredients did not interfere in the assay of NF by the proposed methods. According to the student's *t*- and Variance ratio *F*-tests, there were no significant differences between the calculated and theoretical values at p=0.05, demonstrating that the proposed methods are as accurate and precise as the simple conventional UV-spectrophotometric method (15).

**Table 4:** Assay results for the determination of NF in Praxilene^®^ tablets^a^ using the derivative-spectrophotometric methods

Sample No	Mean % Found^b^ (RSD (%))						
	Measurement						Reference method^c^
	^1^D_237_ (NF)	^1^D_263–299_ (NF)	^2^D_241_ (NF)	^2^D_282–311_ (NF)	^1^D_275_ (PHN)	^2^D_248–263_ (PHN)	

1	100.80 (0.39)	99.00 (0.59)	99.45 (1.35)	98.12 (1.55)	102.40 (1.95)	101.66 (1.22)	
2	99.00 (0.51)	98.70 (0.29)	98.89 (1.50)	97.36 (2.03)	103.00 (1.46)	102.82 (1.00)	
3	99.50 (0.30)	100.50 (0.95)	100.50 (0.96)	100.80 (1.98)	96.50 (0.57)	97.50 (2.00)	
4	101.50 (1.22)	101.00 (1.45)	101.15 (1.45)	100.95 (1.41)	99.40 (0.33)	99.21 (1.48)	
5	102.00 (1.50)	97.50 (1.32)	103.37 (1.90)	96.56 (1.25)	98.90 (0.16)	98.00 (1.34)	
Mean	100.56	99.34	100.67	98.76	100.04	99.84	99.87
RSD (%)	1.27	1.42	1.74	1.85	2.04	2.32	1.99
t^d^	0.648	0.489	0.674	0.883	0.111	0.026	
F^d^	2.406	1.973	1.295	1.023	1.807	1.357	

a Praxilene^®^ tablets are labeled to contain 200 mg per tablet;

b Mean % Found of five determinations;

c Measurement of A_max_ in saline at 283 nm (15);

d Theoretical values for *t* and *F* at p = 0.05 are 2.31 and 6.39, respectively, for n = 5.

**Table 5:** Assay results for the determination of NF in Praxilene^®^ tablets^a^ using the spectrofluorimetric and DP voltammetric methods

Sample No	Mean % Found^b^ (RSD (%))			
	Spectrofluorimetry		DP Voltammetry	Reference method c
	(NF) λ_ex/em_ (277/331 nm)	(PHN) λ_ex/em_ (258/385 nm)	(PHN) at −1000 mV	

1	101.56 (2.07)	98.40 (0.61)	98.10 (0.51)	
2	100.85 (0.64)	97.70 (0.88)	99.20 (1.41)	
3	103.00 (1.48)	99.70 (0.75)	101.30 (2.00)	
4	100.37 (0.39)	98.45 (1.28)	98.20 (1.22)	
5	100.88 (0.29)	101.50 (0.26)	97.53 (2.02)	
Mean	101.33	99.15	98.87	99.87
RSD (%)	1.01	1.51	1.50	1.99
t^d^	1.457	0.65	0.908	
F^d^	3.768	1.761	1.786	

a Praxilene^®^ tablets are labeled to contain 200 mg per tablet;

b Mean % Found of five determinations;

c Measurement of A_max_ in saline at 283 nm (15);

d Theoretical values for *t* and *F* at p = 0.05 are 2.31 and 6.39, respectively, for n=5.

## CONCLUSION

In this study, simple, direct and reliable derivative spectrophotometric, and spectrofluorimetric procedures were developed for the analysis of the vasodilator drug naftidrofuryl oxalate in its dosage form. The chemistry of the condensation reaction of NF with concentrated sulfuric acid was studied and exploited in the development of indirect sensitive derivative spectrophotometric, spectrofluorimetric and voltammetric procedures for the estimation of NF. Reviewing the literature exposed that there were no reports for the polarographic, voltammetric or derivative spectrophotometric determination of the drug. Moreover, few previous studies reported phosphorimetric or spectrophotometric methods for the estimation of NF. The simplicity, convenience at low cost and sensitivity of the proposed methods especially the fluorescence-based are superior or comparable to those of the official non-aqueous titration method (1) and several previously published methods (5, 8–12, 14, 15). In addition to sensitivity, the proposed fluorescence-based and voltammetric methods offer selectivity advantage. Furthermore, the proposed methods do not require elaborate treatment or sophisticated experimental setup usually associated with HPLC methods of analysis. The applicability of the developed methods was evaluated through the determination of NF in bulk form and in pharmaceutical formulation with good accuracy and precision, therefore they can be considered useful and convenient for the routine and quality control assay of the drug.
